# *Sarcina ventriculi* Revisited: A Rare Organism in the Setting of Gastric Dysmotility After Esophagectomy

**DOI:** 10.1155/crdi/8614596

**Published:** 2026-04-07

**Authors:** Sadaf Haiyat, Shabana Azad, Shashikant Patne

**Affiliations:** ^1^ Department of Oncopathology, Mahamana Pandit Madan Mohan Malviya Cancer Centre/Homi Bhabha Cancer Hospital, Homi Bhabha National Institute, Varanasi, India, hbni.ac.in

**Keywords:** delayed gastric emptying, esophagectomy, gastric infection, gastric stasis, histopathology, *Sarcina ventriculi*

## Abstract

**Background:**

*Sarcina ventriculi* is a rare gram‐positive, obligate anaerobic coccus that thrives in acidic environments and has been increasingly recognized in association with delayed gastric emptying and gastric stasis. Although often considered an incidental finding, it has been linked to clinically significant complications, including emphysematous gastritis and gastric perforation Ratuapli S. K. (2013), Laass M. W. (2010), Tolentino L. F. (2003), Lam‐Himlin D. (2011), Al Rasheed M. R. H. (2016). Recognition of this organism is important because identification relies primarily on histologic examination and may directly influence patient management.

**Case Presentation:**

We report a 35‐year‐old male with a history of poorly differentiated adenocarcinoma of the lower esophagus treated with neoadjuvant chemotherapy followed by Ivor–Lewis esophagogastrectomy. Several months after surgery, he developed dysphagia, abdominal discomfort, and symptoms suggestive of impaired gastric emptying. Endoscopic evaluation demonstrated mucosal inflammation and retained food material at the anastomotic site. Histopathologic examination of biopsy specimens revealed inflamed gastric‐type mucosa containing characteristic tetrad and octet packet formations consistent with *Sarcina ventriculi*, without evidence of recurrent malignancy. The histologic diagnosis was communicated to the treating team and directly guided initiation of targeted antimicrobial therapy with metronidazole in combination with proton pump inhibitors and prokinetic agents. The patient demonstrated significant clinical improvement and remained stable on follow‐up.

**Conclusion:**

This case highlights the importance of recognizing *Sarcina ventriculi* in patients with surgically altered gastrointestinal anatomy and delayed gastric emptying. Histologic identification can have direct therapeutic implications and underscores the need for multidisciplinary communication. Increased awareness of this rare but clinically relevant organism may help prevent potential complications and optimize patient outcomes.

**Highlights**
• *Sarcina ventriculi* is associated with delayed gastric emptying and altered anatomy.
• Postesophagectomy gastric dysmotility predisposes to colonization.
• Histologic recognition directly influences antimicrobial management.

## 1. Introduction

*Sarcina* *ventriculi* is a gram‐positive, nonmotile, obligate anaerobic coccus that thrives in acidic environments and ferments carbohydrates to produce carbon dioxide and ethanol. First described by Goodsir in 1842, it has been sporadically reported in human gastric biopsies and is characterized histologically by its distinctive cuboidal morphology arranged in tetrads or octets [1–4].

Although sometimes encountered as an incidental finding, *S. ventriculi* has been increasingly linked to conditions associated with delayed gastric emptying, including gastroparesis, gastric outlet obstruction, and bezoar formation [1, 2, 5, 6]. More aggressive manifestations such as emphysematous gastritis, gastric perforation, and fatal outcomes have also been reported. [7–9]. The pathogenic role of the organism remains controversial, and its detection relies primarily on histomorphologic recognition [1, 2].

Most previously reported cases involve primary gastric motility disorders or mechanical obstruction. In contrast, surgically altered anatomy following esophagectomy represents a distinct mechanism predisposing to gastric stasis and subsequent *Sarcina ventriculi* colonization. The present report documents this rare association and emphasizes the importance of multidisciplinary communication between pathologists and clinicians for appropriate patient management [1, 2].

Our findings emphasize the need for heightened awareness among pathologists to recognize its distinctive morphology and correlate its presence with clinical and endoscopic features, thereby preventing misinterpretation and underreporting of this unusual but important organism.

## 2. Case Presentation

A 35‐year‐old male presented with progressive dysphagia and weight loss and was diagnosed with poorly differentiated adenocarcinoma of the lower esophagus on endoscopic biopsy. He received neoadjuvant neoadjuvant chemotherapy with a regimen comprising 5‐fluorouracil, docetaxel, and oxaliplatin followed by Ivor–Lewis esophagogastrectomy. The postoperative course was uneventful, and adjuvant chemotherapy was completed.

Several months after surgery, during routine follow‐up, the patient developed dysphagia, abdominal discomfort, and symptoms suggestive of impaired gastric emptying. Imaging studies demonstrated inflammatory changes at the anastomotic site without evidence of recurrent or metastatic disease. Upper gastrointestinal endoscopy revealed grade B esophagitis with mucosal erythema, edema, and retained food material, raising suspicion for delayed gastric emptying. Multiple biopsies were obtained from the anastomotic region.

**FIGURE 1:**
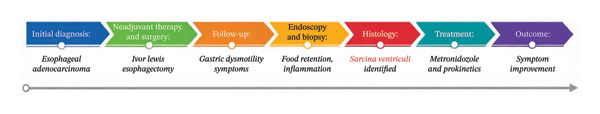
Clinical timeline and diagnostic workflow of the patient.

Histopathological examination revealed inflamed gastric‐type mucosa with granulation tissue and dense neutrophilic infiltrate. Numerous basophilic cocci arranged in characteristic tetrads and octets consistent with *Sarcina ventriculi* were identified. No evidence of recurrent carcinoma was seen. The histologic identification of *S. ventriculi* was communicated to the treating team and directly influenced clinical management, prompting initiation of targeted antimicrobial and prokinetic therapy. The chronological sequence of clinical events is summarized in (Table 1). The clinical course and diagnostic workflow are also illustrated in (Figure 1).

**TABLE 1:** Timeline of clinical events.

1	Patient presented with progressive dysphagia and weight loss; endoscopic biopsy confirmed poorly differentiated adenocarcinoma of the lower esophagus.
2	Neoadjuvant chemotherapy administered, followed by Ivor–Lewis esophagogastrectomy.
3	Postoperative recovery was uneventful; adjuvant chemotherapy completed.
4	During routine follow‐up several months after surgery, the patient developed dysphagia, abdominal discomfort, and symptoms suggestive of delayed gastric emptying.
5	Radiologic evaluation showed inflammatory changes at the anastomotic site without evidence of recurrent or metastatic disease.
6	Upper gastrointestinal endoscopy revealed mucosal inflammation and retained food material at the anastomotic site; biopsies were obtained.
7	Histopathological examination identified *Sarcina ventriculi* in inflamed gastric‐type mucosa with no evidence of recurrent malignancy (Table 1).
8	Findings were communicated to the treating team; targeted therapy with antibiotics, proton pump inhibitors, and prokinetic agents was initiated.
9	Patient showed significant symptomatic improvement and remained clinically stable on follow‐up.

## 3. Microscopic Findings

**FIGURE 2:**
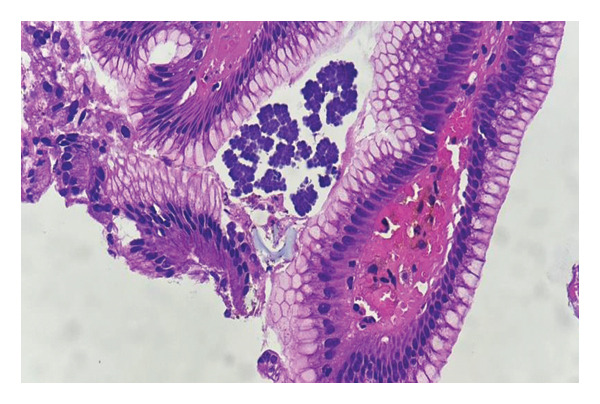
Low‐power photomicrograph (H&E, × 10) showing inflamed gastric‐type mucosa from the anastomotic region with surface debris containing characteristic basophilic, cuboidal bacterial packets of *Sarcina ventriculi* arranged in tetrads.

**FIGURE 3:**
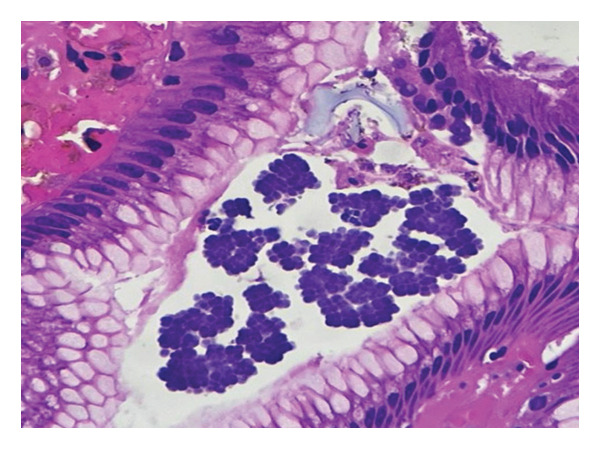
High‐power photomicrograph (H&E, × 40) highlighting *Sarcina ventriculi* appearing as densely packed tetrads and octets along the mucosal surface, with associated acute inflammatory infiltrate and no evidence of recurrent malignancy.

Biopsy fragments from the anastomotic region showed inflamed gastric‐type mucosa with surface debris and dense acute inflammatory infiltrate. Numerous cuboidal bacterial packets arranged in tetrads and octets, characteristic of *S*. *ventriculi*, were present along the mucosal surface. No dysplasia or recurrent invasive malignancy was identified (Figures 2 and 3).

## 4. Treatment and Follow‐Up

The patient received antibiotic therapy with metronidazole along with proton pump inhibitors and prokinetic agents. His gastrointestinal symptoms improved significantly, and he remained clinically stable on subsequent follow‐up evaluations. No further endoscopic or radiologic evidence of disease recurrence was identified.

## 5. Discussion

*Sarcina ventriculi* is a rare anaerobic organism with distinctive histologic features that allow diagnosis on routine hematoxylin‐ and eosin‐stained sections. Its packet‐like morphology results from division in two perpendicular planes. Despite its characteristic appearance, the organism may be overlooked or misinterpreted as vegetable matter, emphasizing the need for diagnostic vigilance [1, 10].

The clinical relevance of *S. ventriculi* remains debated. While some cases are indolent, others have been associated with severe complications such as emphysematous gastritis, gastric perforation, and fatal outcomes [7–9, 11]. Most reported cases involve conditions causing gastric stasis, including gastroparesis or gastric outlet obstruction [1, 2, 6]. In postesophagectomy patients, altered anatomy and impaired gastric motility create a unique environment favoring bacterial overgrowth, representing a distinct pathogenic mechanism [10].

Comparison of previously reported cases highlights distinct clinical contexts in which *Sarcina ventriculi* is encountered, including primary gastric motility disorders, mechanical gastric outlet obstruction, and surgically altered anatomy (Table 2). In patients with primary gastroparesis, chronic functional delay in gastric emptying creates a permissive environment for *Sarcina* colonization, typically resulting in an indolent clinical course. In contrast, cases associated with gastric outlet obstruction often exhibit more severe presentations due to mechanical stasis and carry a higher risk of complications such as emphysematous gastritis or perforation.

**TABLE 2:** Comparison of *Sarcina ventriculi* cases across different clinical settings.

Feature	Primary gastroparesis	Gastric outlet obstruction	Surgically altered anatomy (present case)
Underlying mechanism	Functional delay in gastric emptying due to autonomic or neuromuscular dysfunction	Mechanical obstruction secondary to pyloric stenosis, tumors, or bezoars	Postesophagectomy anatomical alteration with impaired gastric reservoir function and motility
Typical patient population	Patients with diabetic or idiopathic gastroparesis	Patients with peptic ulcer disease, gastric malignancy, or bezoars	Postesophagectomy patient
Predisposing factor for *S. ventriculi*	Chronic functional gastric stasis	Obstructive gastric stasis	Combined anatomical disruption and delayed gastric emptying
Mode of diagnosis	Histologic identification on gastric biopsy	Histologic identification on gastric biopsy	Histologic identification on anastomotic biopsy
Clinical course	Typically indolent; may be symptomatic	Variable; may be severe	Symptomatic but clinically stable
Treatment approach	Antibiotics ± proton pump inhibitors; prokinetic agents	Antibiotics with relief of mechanical obstruction	Antibiotics, proton pump inhibitors, and prokinetic agents
Reported complications	Rare but reported	Increased risk of emphysematous gastritis and gastric perforation	No complications observed

The present case represents a third and less frequently described category, in which postesophagectomy anatomical alteration and impaired gastric reservoir function collectively predisposed to delayed gastric emptying and *Sarcina* proliferation. Notably, unlike obstruction‐related cases, our patient demonstrated a stable but symptomatic course without life‐threatening complications. This comparative framework underscores the importance of recognizing surgically altered anatomy as a distinct risk setting and supports a tailored management strategy based on the underlying mechanism of gastric stasis rather than the mere presence of the organism (Table 2).

There are no standardized treatment guidelines for *Sarcina ventriculi*; however, most cases respond to metronidazole‐based therapy with acid suppression, while surgery is reserved for severe complications [2, 5, 10]. Case reports describe successful conservative management with metronidazole and proton pump inhibitors in patients with delayed gastric emptying or gastroparesis [7, 8].

Published literature demonstrates a consistent association between *Sarcina ventriculi* infection and conditions causing gastric stasis, with management strategies largely guided by disease severity (Table 3). Laass et al. reported symptomatic improvement in a patient with delayed gastric emptying and gastric ulcer following treatment with metronidazole and proton pump inhibitors [7]. Tolentino et al. described cases associated with gastroparesis that responded favorably to conservative metronidazole‐based therapy, with or without acid suppression [8]. In a systematic review of 66 cases, Sharma et al. identified gastric stasis or obstruction as the predominant predisposing factors; antimicrobial therapy, most commonly metronidazole alone or combined with fluoroquinolones, was effective in many patients, while surgical intervention was required in severe cases, with an overall mortality of 14% [10]. Fulminant presentations such as emphysematous gastritis and gastric perforation have been reported, necessitating aggressive management with broad‐spectrum antibiotics and, in some cases, surgical intervention to improve survival outcomes [9, 11]. In contrast, the present case highlights a more indolent presentation in the setting of surgically altered anatomy, with clinical improvement achieved using metronidazole, acid suppression, and prokinetic agents, reinforcing the importance of early recognition and tailored therapy (Table 3).

**TABLE 3:** Literature review of previously published cases of *Sarcina ventriculi* with clinical setting and treatment approaches.

Author (year)	Clinical setting	Predisposing factor	Treatment regimen	Outcome
Laass et al. (2011)	Gastric ulcer	Delayed gastric emptying	Metronidazole + PPI	Symptomatic improvement
Tolentino et al. (2013)	Gastroparesis	Functional gastric stasis	Metronidazole ± PPI	Clinical resolution
Sharma et al. (2021)	Systematic review (66 cases)	Gastric stasis/obstruction	Metronidazole ± fluoroquinolone; surgery in severe cases	14% mortality; improvement in survivors
Singh et al. (2020)	Emphysematous gastritis	Severe gastric stasis	Broad‐spectrum antibiotics + surgery	Survival with intervention
Present case	Surgically altered anatomy	Combined anatomical disruption and delayed gastric emptying	Broad‐spectrum antibiotics + PPI + Prokinetics	Survival with intervention

While histopathologic evaluation remains the primary diagnostic modality, adjunct molecular methods have been reported and may assist in selecting diagnostically difficult cases. PCR‐based assays targeting 16S rRNA gene sequences have been utilized to confirm *Sarcina ventriculi*, particularly when organisms are sparse, morphology is atypical, or background debris limits histologic assessment. Such molecular approaches may also help distinguish *S. ventriculi* from histologic mimics, including vegetable material and other anaerobic cocci. Nevertheless, these techniques are not routinely implemented in diagnostic pathology laboratories, lack standardized validation, and are largely confined to research applications or highly selected clinical contexts [1].

The novelty of this report lies in the identification of *Sarcina ventriculi* in a patient who developed dysphagia following esophagogastrectomy for esophageal carcinoma. Detection of *S. ventriculi* in a surgically altered gastric environment underscores its potential role not only as an indicator of impaired gastric motility but also as a possible contributor to mucosal injury and ulcer formation. Notably, in contrast to most published reports that emphasize fulminant complications such as gastric perforation, the present case demonstrates a more indolent yet clinically meaningful presentation.

This case highlights two important considerations. First, diagnostic vigilance is essential, as the organism’s characteristic morphology may be misinterpreted as vegetable matter or overlooked, resulting in underrecognition. Second, altered postoperative anatomy and physiology likely predisposed this patient to gastric stasis, creating a favorable environment for *Sarcina* colonization and proliferation.

Collectively, this report broadens the clinicopathologic spectrum of *S. ventriculi* and emphasizes the importance of heightened awareness among both pathologists and clinicians. Recognition of this organism on histologic examination should prompt evaluation for delayed gastric emptying, particularly in postsurgical contexts, and may serve as an early indicator of potential downstream complications.

## 6. Conclusion

*Sarcina ventriculi* is an uncommon but clinically significant organism that should be recognized in gastric biopsies, particularly in patients with surgically altered gastrointestinal anatomy. Its identification has direct implications for patient management and may serve as a marker of delayed gastric emptying. Increased awareness among pathologists and clinicians is essential to prevent underdiagnosis and potential complications.

## Author Contributions

Sadaf Haiyat conceptualized the study, interpreted the histopathological findings, and prepared the manuscript. Shabana Azad contributed to clinical data collection and literature review. Shashikant Patne provided critical revision and final approval of the manuscript.

## Funding

This research received no external funding.

## Disclosure

This​ case report has been prepared in accordance with the CARE (CAse REport) guidelines. The completed CARE checklist is provided as Supporting Information.

This work has not been previously published as a preprint and has not been presented at any scientific conference or seminar. All the authors approved the final version.

## Ethics Statement

Institutional Review Board approval was not required and therefore not obtained for this case report, in accordance with institutional policies for case reporting.

## Consent

In accordance with institutional policy, formal written informed consent was waived for this case report. The report contains no identifiable patient information, and all data have been fully anonymized to protect patient confidentiality.

## Conflicts of Interest

The authors declare no conflicts of interest.

## Data Availability Statement

All data generated or analyzed during this study are included in this published article.

## References

[1] Lam-Himlin D. , Tsiatis A. C. , Montgomery E. et al., *Sarcina* Organisms in the Gastrointestinal Tract: A Clinicopathologic and Molecular Study, American Journal of Surgical Pathology. (2011) 35, no. 11, 1700–1705, 10.1097/pas.0b013e31822911e6, 2-s2.0-80055015206. 21997690 PMC3193598

[2] Al Rasheed M. R. H. and Senseng C. G. , *Sarcina ventriculi*: Review of the Literature, Archives of Pathology and Laboratory Medicine. (2016) 140, no. 12, 1441–1445, 10.5858/arpa.2016-0028-rs, 2-s2.0-85004098358. 27922772

[3] Goodsir J. and Wilson G. , History of a Case in Which a Fluid Periodically Ejected From the Stomach Contained Vegetable Organisms of an Undescribed Form, Edinburgh Medical and Surgical Journal. (1842) 57, no. 151, 430–443. PMC5791290 30330668

[4] Beijerinck M. W. , An Experiment With *Sarcina ventriculi* , Proceedings of the Royal Netherlands Academy of Arts and Sciences. (1911) 13, 1234–1240.

[5] Ratuapli S. K. , Lam-Himlin D. , and Heigh R. I. , *Sarcina ventriculi* of the Stomach: A Case Report, World Journal of Gastroenterology. (2013) 19, no. 14, 2282–2285, 10.3748/wjg.v19.i14.2282, 2-s2.0-84876349527. 23599657 PMC3627895

[6] Sopha S. C. , Kwon Y. , Danford C. J. et al., *Sarcina* in the Bariatric Era, Human Pathology. (2015) 46, 1405–1410. 26198746 10.1016/j.humpath.2015.05.021

[7] Laass M. W. , Pargac N. , Fischer R. , Bernhardt H. , Knoke M. , and Henker J. , Emphysematous Gastritis Caused by *Sarcina ventriculi* , Gastrointestinal Endoscopy. (2010) 72, no. 5, 1101–1103, 10.1016/j.gie.2010.02.021, 2-s2.0-78049321988. 20538273

[8] Tolentino L. F. , Kallichanda N. , Javier B. , Yoshimori R. , and French S. W. , Gastric Perforation Associated With *Sarcina ventriculi* , Laboratory Medicine. (2003) 34, 535–537, 10.1309/cdff04he9fhdqpan, 2-s2.0-0038648827.

[9] Brown I. S. and Bettington M. , *Sarcina ventriculi* Revisited, Pathology. (2021) 53, 589–595.

[10] Sharma A. , Lam-Himlin D. , and Brown I. S. , *Sarcina ventriculi* in Surgically Altered Gastrointestinal Anatomy: A Systematic Review of Reported Cases, Annals of Diagnostic Pathology. (2021) 52.

[11] Dumitru A. , Alius C. , Popescu I. et al., Fatal Outcome of Gastric Perforation due to *Sarcina* spp, IDCases. (2020) 19. 10.1016/j.idcr.2020.e00711 PMC7031000 32099809

